# Applications of Chinese *Camellia oleifera* and its By-Products: A Review

**DOI:** 10.3389/fchem.2022.921246

**Published:** 2022-05-24

**Authors:** Wenxuan Quan, Anping Wang, Chao Gao, Chaochan Li

**Affiliations:** ^1^ Guizhou Provincial Key Laboratory for Information Systems of Mountainous Areas and Protection of Ecological Environment, Guizhou Normal University, Guiyang, China; ^2^ Key Laboratory of Forest Cultivation in Plateau Mountain of Guizhou Province, Institute for Forest Resources and Environment of Guizhou, Guizhou University, Guiyang, China

**Keywords:** camellia oil, by-products, medical value, activated carbon, applications

## Abstract

*Camellia oleifera* is a woody oil tree species unique to China that has been cultivated and used in China for more than 2,300 years. Most biological research on *C. oleifera* in recent years has focused on the development of new varieties and breeding. Novel genomic information has been generated for *C. oleifera*, including a high-quality reference genome at the chromosome level. Camellia seeds are used to process high-quality edible oil; they are also often used in medicine, health foods, and daily chemical products and have shown promise for the treatment and prevention of diseases. *C. oleifera* by-products, such as camellia seed cake, saponin, and fruit shell are widely used in the daily chemical, dyeing, papermaking, chemical fibre, textile, and pesticide industries. *C. oleifera* shell can also be used to prepare activated carbon electrodes, which have high electrochemical performance when used as the negative electrode of lithium-ion batteries. *C. oleifera* is an economically valuable plant with diverse uses, and accelerating the utilization of its by-products will greatly enhance its industrial value.

## Introduction

Edible oil is an important food for humans that provides essential fatty acids and promotes the absorption of fat-soluble vitamins (Soussanaet 2014; Khatri and Jain, 2017). China is the world’s largest consumer and second-largest producer of edible oil (Cassiday 2019; Bai et al., 2021), and the demand for edible oil in China continues to increase with the continued growth of the economy and improvement in living standards.

Edible vegetable oils in China include rapeseed oil, soybean oil, peanut oil, cottonseed oil, sunflower oil, sesame oil, camellia oil, and linseed oil (Bai et al., 2021). Vegetable oils are rich in nutrients and provide various health benefits: camellia oil in particular shows antibacterial activity against *Escherichia coli* (Yang et al., 2018).

**FIGURE 1:**
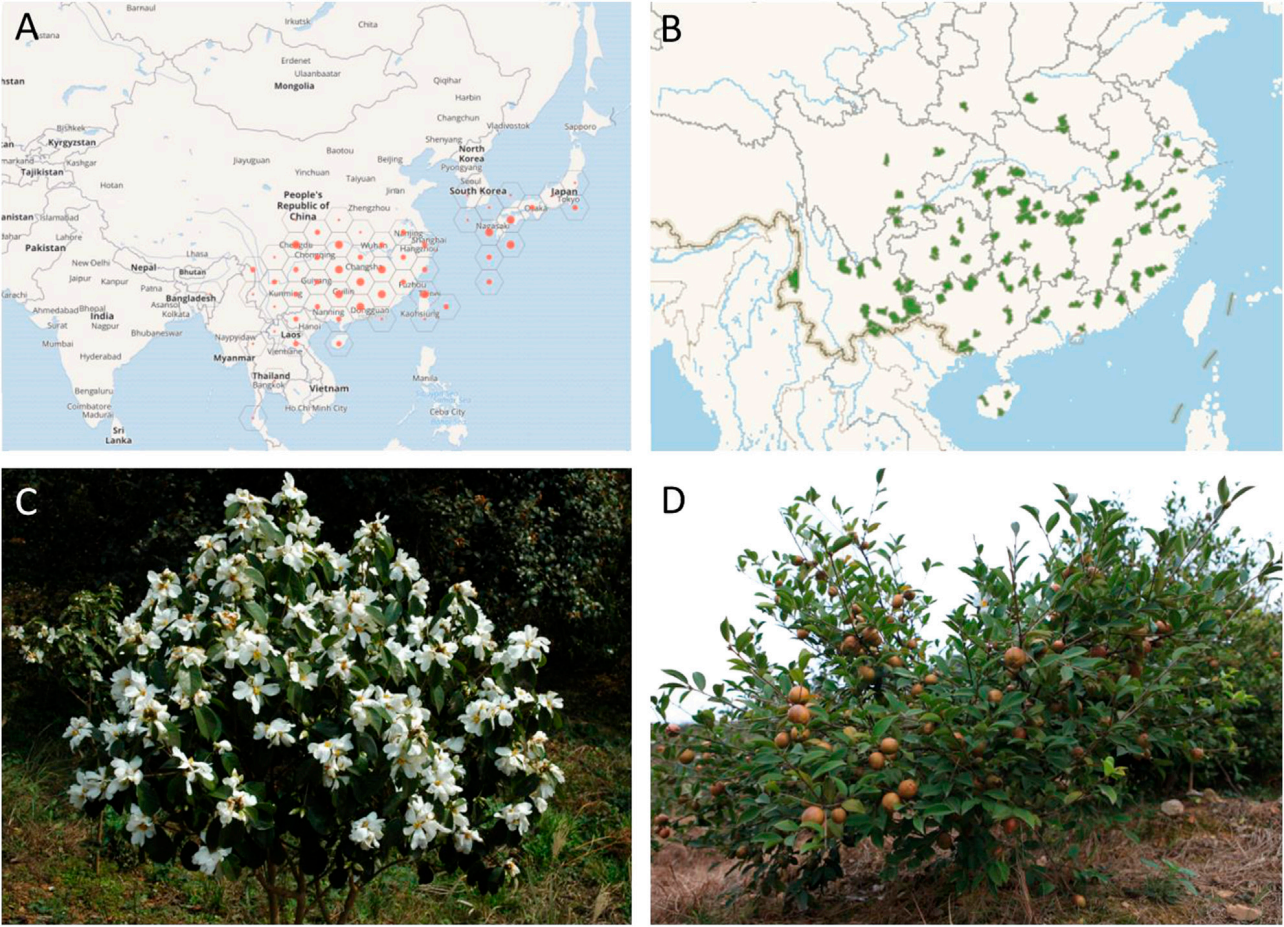
Distribution **(A,B)** of *C. oleifera* and images of the flowers and fruits **(C,D)** of *C. oleifera* plants.

*Camellia oleifera* Abel. is one of the four major sources of the world’s edible oil, along with *Olea europaea* L., *Elaeis guineensis* Jacq., and *Cocos nucifera* L. (Ma et al., 2011). It is a perennial shrub or small arbour that grows in warm and humid hills and mountains and is mainly distributed in the southern provinces (regions) of China (Figure 1), including Zhejiang, Jiangxi, Henan, Hunan, and Guangxi Provinces; it also occurs in Thailand (Suealek et al., 2019). The varieties mainly include ordinary *Camellia oleifera*, *Camellia yuhsiensis* Hu, *Camellia chekangoleosa* Hu, *Camellia meiocarpa* Hu, Camellia vietnamensis T. C. Huang ex Hu, and *Camellia reticulata* Lindl (Liu et al., 2018a). Zhou et al. indicated that the germplasms of *C oleifera* possess high genetic diversity, as geographic isolation has affected the degree of genetic differentiation among populations (Zhou et al., 2015).

*C. oleifera* trees are evergreen and highly adaptable. The benefits of planting *C. oleifera* can be reaped for as long as a century. The total output value of the Chinese camellia industry was 116 billion yuan in 2019, and the plantation area of *C. oleifera* was 4.5 million hm^2^; the camellia industry is a source of income for a total of 1.73 million people.

Figure 1A,B were obtained from the Global Biodiversity Information Facility (https://www.gbif.org/) and Map Bio of China (http://map.especies.cn/), respectively.

*C. oleifera* is an economically important tree species with high utilization value. The main product derived from *C. oleifera* is camellia oil, and other by-products include tea shell and tea meal. Tea meal can be further processed into tea saponin, and tea shell has been used to make furfural, xylitol, tannin extract, activated carbon, and culture medium (Robards et al., 2009).

## Research on the Biology of *C. oleifera*

*C. oleifera* is a woody oil plant that is highly resistant to various types of stress. However, genetic and genomic information for this species is lacking (Yang et al., 2017). The large polyploid genome of *C. oleifera* makes genomic analyses rather challenging and hinders further molecular genetic improvement. Recently, an abundance of genomic information has been generated for *C. oleifera*. The published genome of *Camellia lanceoleosa* provides an important reference for analyzing the formation and regulation of important traits such as self-incompatibility and lipid synthesis (Gong et al., 2022). Construction of a high-quality reference genome at the chromosome level of *C. oleifera* has demonstrated that the alleles regulating the synthesis of *C. oleifera* have been under artificial selection, and this genome resource could provide new insights with implications for the genetic improvement of *C. oleifera* varieties (Lin et al., 2022). In addition, a high-quality, chromosome-level genome of *Camellia chekiangoleosa* has been published, and this has provided new insights into the adaptive evolution and oil metabolism of Camellia (Shen et al., 2022).

Wang et al. (2018) found that the total nitrogen content and dry weight accumulation of the seedlings are highest when NO_3_^−^ and NH_4_^+^ (ratio 1:1) are applied. Liu et al. (2019b) found that a total of 797 miRNAs are significantly differentially expressed in the flowers and fruits of *C. oleifera*. miR156, miR390, and miR395 regulate the expression of carbohydrate accumulation genes, and miR477 plays a key role in fatty acid synthesis. miR156 contributes to the expression of genes regulating glycolysis and nutrient transformation.

The high rate of flower and fruit drop in *C. oleifera*, especially under extreme climate conditions, affects *C. oleifera* yields. Hu et al. (2021) studied the relationship between ethylene and fruit abscission and found that the CoACO genes (*CoACO1* and *CoACO2*) regulate fruit abscission.

*C. oleifera* is highly tolerant of drought. An understanding of the molecular mechanism of drought tolerance is important. Dong et al. (2017) identified several 76,585 unigenes under drought stress using transcriptome technology and obtained functional annotations for 52,531 of the unigenes.

**FIGURE 2:**
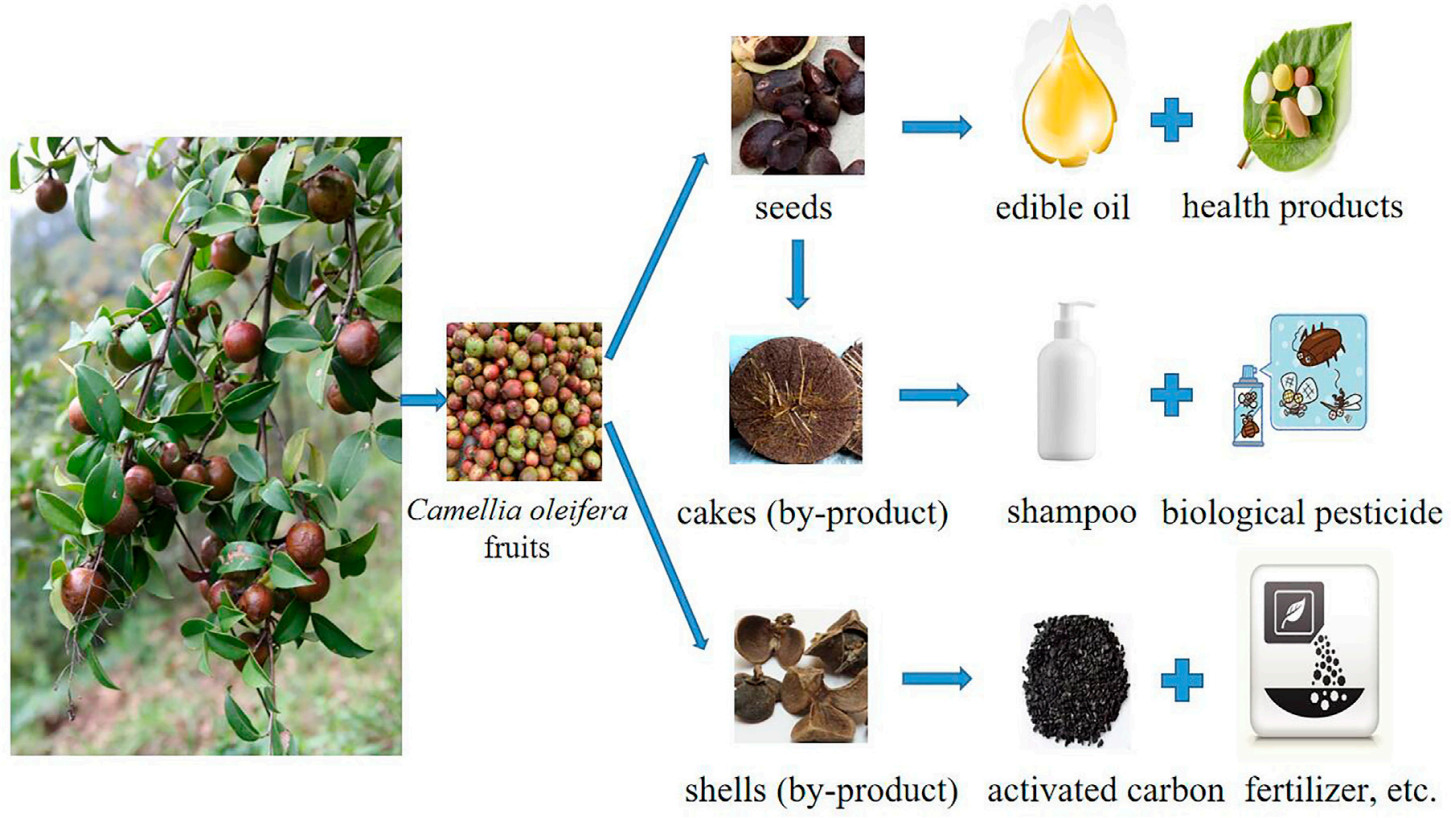
Diagram illustrating the various industrial uses of *C. oleifera*.

Other studies have examined the high-affinity Pi transporter gene and have characterized *rbcL* and *rbcS* genes from *C. oleifera* (Chen et al., 2015; Zhou et al., 2020). These findings are useful for identifying promising cultivars (Figure 2)

## Camellia Oil and the Composition of its Main Fatty Acids

The oil content of the dry seeds of new cultivars and wild *C. oleifera* is approximately 47%; the dry seeds also possess volatile aroma components (Jia et al., 2021). The content of unsaturated fatty acids of *C. oleifera* oil is as high as 90%; oleic acid makes up more than 80% of these unsaturated fatty acids, and linoleic acid comprises 7–13% (Ma et al., 2011; Yang et al., 2016). Variation in the composition of unsaturated fatty acids mainly stems from differences in genotype and extraction method (Zeng et al., 2019a) (Table 1). Fatty acids can be extracted using the petroleum ether, hydrolytic, or potassium hydroxide/methanol extraction methods. The fatty acid composition of camellia oil is mainly determined using gas chromatography or gas chromatography–mass spectrometry.

**TABLE 1:** Fatty acid composition of camellia oil according to studies using different extraction methods.

No.	Analytical Method	Main Fatty Acids (% of the Total Fatty Acids)	References
1	Petroleum ether extraction and gas chromatography (GC)	82–84% unsaturated fatty acids (UFA),68–77% monounsaturated fatty acids (MUFA), 7–14% polyunsaturated fatty acids (PUFA).	Ma et al. (2011)
2	gas chromatography–mass spectrometry (GC–MS)	90% UFA, 66.54–83.24% oleic acid, 8.15–9.70% palmitic acid, 5.64–7.96% linoleic acid.	Yuan et al. (2012)
3	GC–MS	87.45–90.17% UFA, 77.08–82.78% MUFA, 5.17–11.27% PUFA.	Yang et al. (2016)
4	Hydrolytic extraction and GC	10–10.4% SFA, 89.55–90.00% UFA, 79.35–81.60% MUFA, 8.40–10.20% PUFA.	Cao et al. (2017)
5	GC	12.65–12.40% SFA, 79.16–81.05% MUFA, 8.19–8.04% PUFA.	Zhang et al. (2019)
6	Methanol extraction and GC–MS	87.85–91.44% UFA, 80.53– 86.18% oleic acid, 6.72–9.26% palmitic acid, 4.19–8.95% linoleic acid, 0.84–1.65% stearic acid, 0.09–0.26% eicosenoic acid	Liu et al. (2021)

Oleic acid provides various health benefits (Farooqui 2013); olive oil is approximately 59–75% oleic acid (Newmark 1997), and palm oil contains 43% oleic acid (Waterman and Lockwood 2007). The main characteristic feature of camellia oil is its high oleic acid content compared with other woody edible oils.

## *C. oleifera* Products

### Medicinal Research on Camellia Oil

Camellia oil contains tocopherol, sterol, squalene, vitamin E, and flavonoids (Lee and Yen 2006; Robards 2009; Cao et al., 2017; Wang et al., 2017; Zeng and Endo 2019b), and these compounds are thought to aid weight loss and reduce the risks of cardiovascular and cerebrovascular diseases.

Camellia oil also contains large amounts of functional nutrients, such as squalene, plant sterols (e.g., β-sitosterol and campesterol), polyphenols (e.g., phenolic acid), tocopherols (α-, γ-, and δ-tocopherols), carotenoids (e.g., lycopene), β-carotene, and lutein (Wang et al., 2017; Wang et al., 2018; Yang et al., 2018). These active substances can delay the degradation of unsaturated fatty acids in camellia oil (Zhou et al., 2019), provide various health benefits (Luan et al., 2020), and show antioxidant, anti-inflammatory, and antibacterial activity (Zhu et al., 2019). These compounds can also lower cholesterol, blood sugar, and blood lipids, relieve constipation, and reduce liver and gastrointestinal damage (Table 2).

**TABLE 2:** Specific medicinal uses of camellia oil.

No.	Materials	Experimental Model	Specific Medicinal Use	References
1	Camellia seed	Male Wistar rats	Repair nonalcoholic fatty liver disease	Yeh et al. (2019)
2	Camellia oil	Male Sprague-Dawley rats	Repair oxidative damage in the stomach and intestine	Cheng et al. (2014)
3	Camellia oil	Male BALB/c mice	Ameliorate ethanol-induced acute gastric mucosal injury	Tu et al. (2017)
4	Camellia oil	Four-week-old male BALB/c mice	Repair gastrointestinal mucosal damage	Wang et al. (2019a)
5	Camellia oil	Human Int-407 cells; Female Sprague-Dawley rats	Mitigate Alzheimer’s disease (AD)	Weng et al. (2020)
6	Camellia oil	Hamsters	Reduce fat	Suealeket al. (2019)
7	Camellia oil	Female ovariectomized mice	Reduce fat	Tung et al. (2019)
8	Camellia seed	Five human cancer cell lines	Anticancer: saponin OSC6 is a potential therapeutic agent for the treatment of cancer	Zong et al. (2016)
9	Camellia seed	Male ICR mice	Anticancer: a new glycoprotein (COG2a) has anticancer action.	Li et al. (2019)
10	Camellia seed	Wistar rats	Hepatoprotective effects	Ko et al. (2019)
11	Camellia oil	Male Sprague-Dawley rats	Alleviates colitis	Lee et al. (2018)
12	Camellia oil	2,2-diphenyl-1-picrylhydrazyl (DPPH) scavenging activity and Trolox equivalent antioxidant capacity	Free radical scavenging: two compounds isolated exhibit antioxidant activity.	Lee and Yen, (2006)

Camellia oil can be used as a nutritional supplement and be further refined and processed into an advanced skin care product. Recently, the seed extract of camellia oil has been shown to reduce liver fat in rats (Yang et al., 2019).

### The Main Nutrient Components of *Camellia* By-Products

Residues such as camellia seed cake, saponin, and fruit shells are widely used in the daily chemical, dyeing, papermaking, chemical fibre, textile, and pesticide industries (Liu et al., 2018b). Previous studies have shown that polysaccharides extracted from fruit shells have hypoglycemic effects (Zhang and Li, 2015; Gao et al., 2020).

The seeds remaining after oil extraction are by-products referred to as camellia seed cake (Xiao et al., 2017). Oil makes up approximately 5%–6% of the seed cake, and the remaining seed cake after extraction can be used to extract 4% of high-quality saponin (Zhu et al., 2018). Recent studies have shown that seed cakes contain large amounts of polyphenols and new saponins with antimelanogenic and hypoglycemic activity (Zhang et al., 2012; Zhang and Li 2018; Hong et al., 2019). For example, kaempferol extracted from seed cakes shows excellent scavenging activity of 2,2-diphenyl-1-picrylhydrazyl (DPPH) radical (Zheng et al., 2019), saponins show anticancer activity (Di et al., 2017; Wang et al., 2019a), and polysaccharides show hypoglycemic activity (Zhang and Li 2018; Zhu et al., 2018; Jin et al., 2019).

Saponin from *C. oleifera* is a natural plant pesticide that shows potential to be used for the control of insect and fungal pests (Zhang et al., 2014). Contact toxicity tests and gastric toxicity tests have shown that saponin is an effective insecticide against *Ectropis obliqua* (Cui et al., 2019). Saponin mixtures can also be used as a potential plant insecticide to control *Rhizoctonia* damping-off in vegetable seedlings (Kuo et al., 2010).

## Utilization of *C. oleifera* Shell

### Morphological Changes of Camellia Shell

**FIGURE 3:**
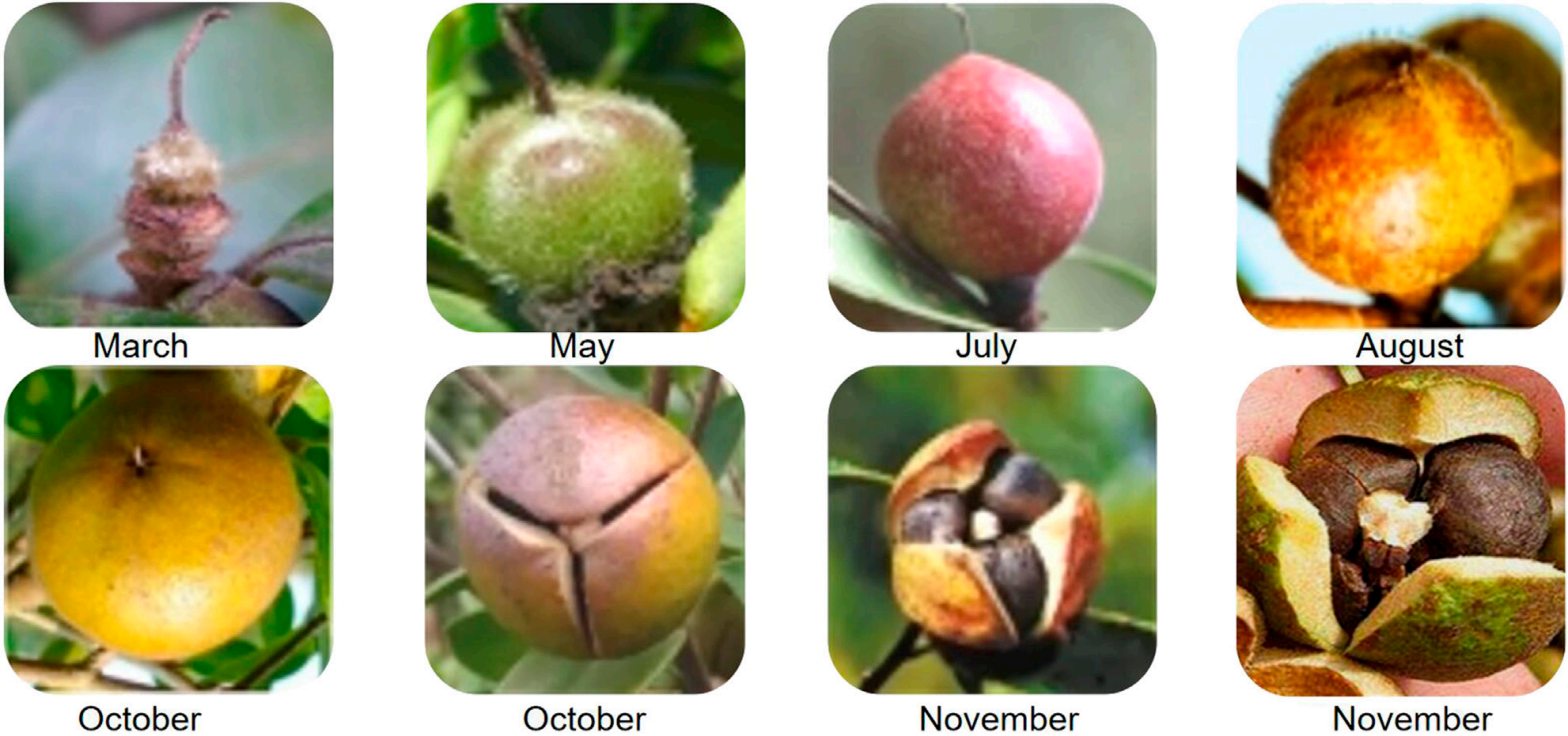
Structural characteristics **(A)** and morphological changes at different stages **(B)** of *C. oleifera* fruit.

*C. oleifera* shell is mainly composed of cellulose, hemicellulose, and lignin; it is generally used as waste given its low utilization efficiency (Hu et al., 2015). Approximately 54% of *C. oleifera* fruits are shells (Zhu et al., 2013; Zhao et al., 2017). Camellia shell is thus a rich biomass resource; the shells contain rich quantities of lignin and are an ideal raw material for preparing activated carbon (Hu et al., 2018). Hu et al. (2018) and Wang et al. (2019b, 2021) showed that mature camellia shell is composed of stone cells, spiral vessels, and parenchyma, and the latter two are the main cell types (Figure 3).

### Functions and Applications of Camellia Shell

The high-quality by-products of *C. oleifera* shell have been used in many industries. Camellia shell contains tannins, furfural, bioactive phenolic compounds (Zhang et al., 2013), and saponins (Chen et al., 2013; Xiong et al., 2018; Yu and Yong 2018), which are used to make tannins, furfural, activated carbon, and other chemical raw materials. Zhu et al. (2013) used camellia shell to produce ethanol, vanillin, and xylooligosaccharides; camellia shell can also be used as a natural colourant for pigment printing on cotton fabrics (Nakpathom et al., 2017).

**FIGURE 4:**
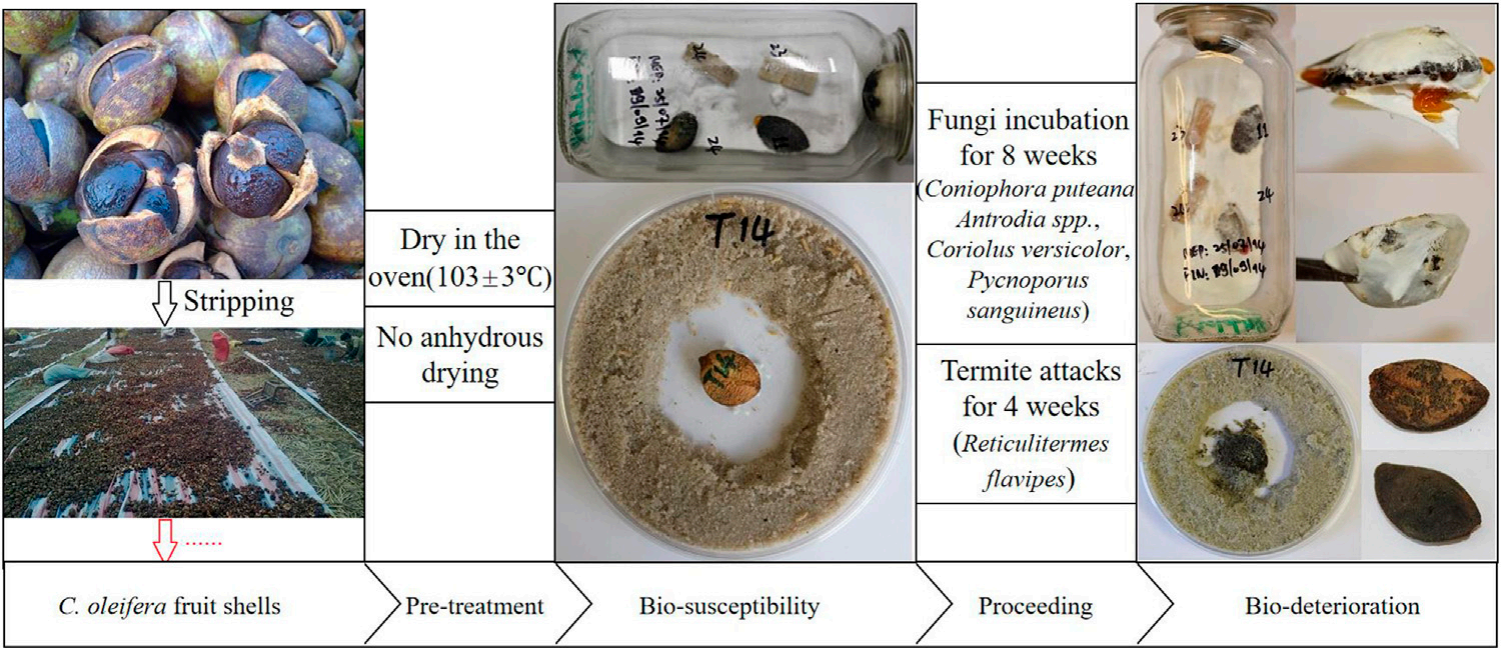
Resistance of camellia shell powder to fungi and termites. Copyright 2015 Elsevier.

Previous studies have examined the ability of camellia shell extract to inhibit tyrosinase activity *in vitro* as well as the melanin inhibition of a cosmetic formula containing the extract in 30 female subjects. Camellia shell extract has been used as a skin whitening agent in cosmetic products (Liu et al., 2019a). Hu et al. (2015) investigated the resistance of camellia shell to fungi and termites; the shells appeared to be toxic to fungi and termites but did not completely eradicate them (Figure 4). This indicates that Camellia shell has the potential to be used as a green pesticide.

### Application of Camellia Shell as High-Quality Activated Carbon

Activated carbon is widely used, and its most notable features are its large surface area, porosity, highly adsorptive internal porous structure, and low cost (Sakaray and Ramirez, 2014). *C. oleifera* shell is composed of cellulose, hemicellulose, and lignin, and highly developed mesoporous activated carbon can be formed through various technologies. The advantages of Camellia shell activated carbon mainly include its high yield and low cost, as well as the fact that it provides a guaranteed source of raw materials; Camellia shell activated carbon also shows high electrical conductivity and can be used as electrodes. Activated carbon has been obtained from many other plant materials, and these activated carbons, such as macadamia nut shell (Wang et al., 2002), *Terminalia catappa* shell (Inbaraj and Sulochana, 2006), peanut shell (Wu et al., 2013), durian fruit shell (Tey et al., 2016), baobab fruit shell (Vunain et al., 2017), *Aegle marmelos* Correa fruit shell (Sivarajasekar et al., 2018), and *Swietenia macrophylla* fruit shell (Hossain et al., 2021), show high application prospects.

Camellia shell activated carbon is a porous carbon material generated through the carbonization and activation process and an economically important chemical product. It shows high selective adsorption and is widely used in decolorization and water purification. Camellia shell activated carbon has more functions compared with the conventional activated carbon, and Camellia shell activated carbon products with different adsorption characteristics can be prepared using various methods (Sun et al., 2011; Guo et al., 2016, 2018; Fan et al., 2017; Nie et al., 2019). *C. oleifera* shell can be used to synthesize zirconium dioxide biochar and improve the removal of fluorine in water (Lei et al., 2019).

**FIGURE 5:**
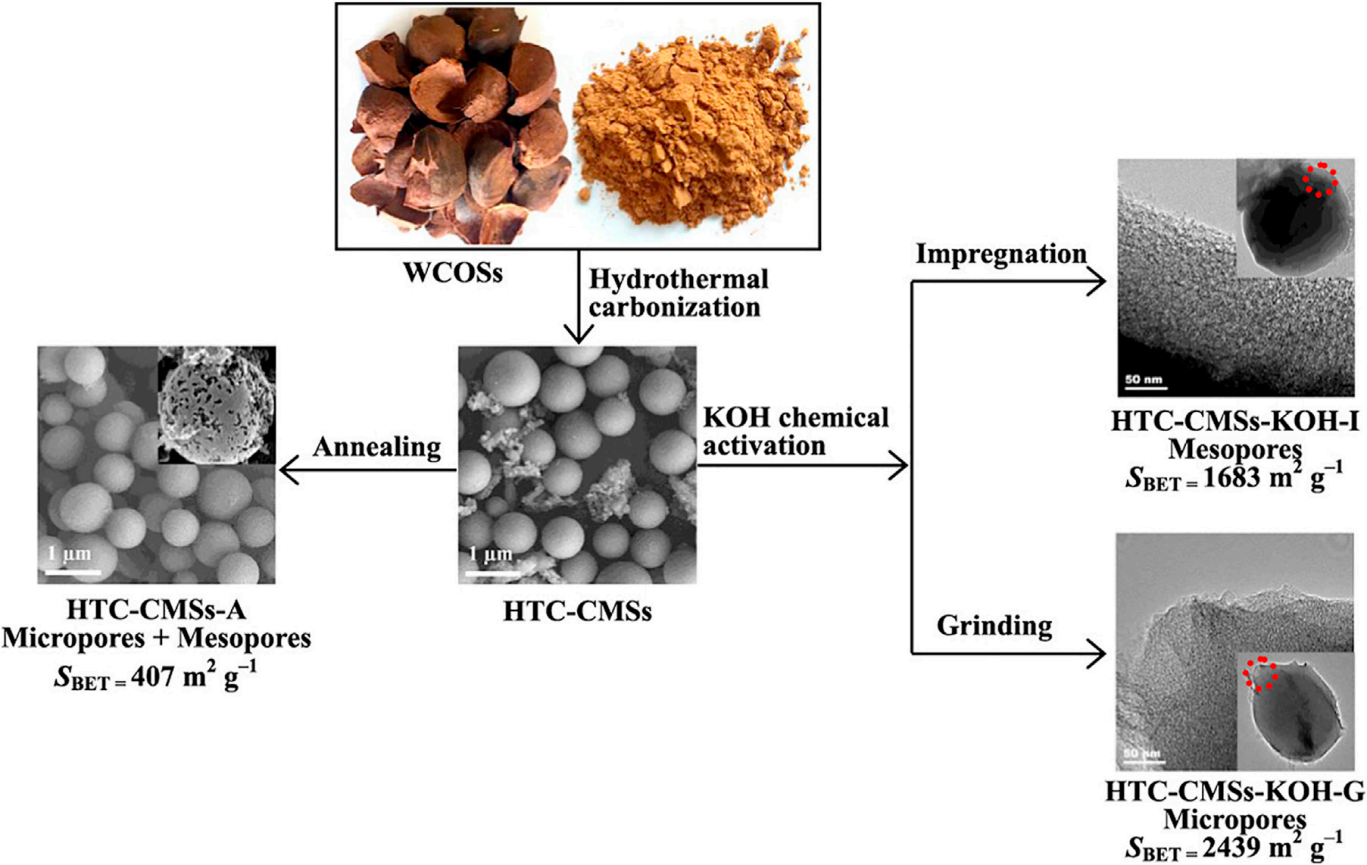
Porous carbon microspheres made from camellia shells by KOH chemical activation. Copyright 2016 Elsevier.

Activated carbon produced by the steam method has many micropores, which is suitable for adsorbing small molecular impurities. The activated carbon produced by the phosphoric acid method has many mesopores and is suitable for adsorbing macromolecular impurities. The phosphoric acid method has become the main method used in the industrial production of activated carbon from husks because it generates fewer pollutants compared with other methods. *C. oleifera* shell carbon can remove hexavalent chromium and methylene blue from water by adsorption (Ma et al., 2019); the shell has similar burning properties to ordinary wood (Tan et al., 2020). It can also rapidly remove phenolic pollutants in water (Figure 5) (Li et al., 2016).

Activated carbon electrodes can be prepared from *C. oleifera* shell, and this is a particularly efficient method for using camellia resources. Zhang et al. (2012) used *C. oleifera* shell to prepare activated carbon electrodes using the ZnCl_2_ activation method. Ma et al. (2019) made a porous carbon material synthesized from *C. oleifera* shells *via* K_2_CO_3_ impregnation and pyrolysis, and this material has excellent electrochemical properties when it is used as the anode of Li-ion batteries. Porous carbon with a three-dimensional porous architecture, large surface area, and electrochemical-active oxygen functionalities has been prepared using the microwave-assisted carbonization/activation method (Liang et al., 2018).

## Conclusion and Prospects

In this review, recent research on *C. oleifera* was summarized, including physiological and ecological research on *C. oleifera* trees, as well as research on the quality and function of camellia oil and the various uses of camellia by-products. The camellia industry has a long industrial chain, and the results of recent research have promoted the development of the entire industrial chain.

*C. oleifera* oil is a high-end edible oil with high medicinal value. The unsaturated fatty acid content of *C. oleifera* oil is greater than 80%. The oil can be used as a nutritional supplement and can also be further refined into an advanced skin care product. Camellia oil can aid weight loss and reduce the risk of cardiovascular and cerebrovascular diseases.

Camellia seed cake has high medicinal value and can be used to develop several products. The seed cake contains a large number of tea polyphenols and saponins, which show anti-melanin, hypoglycemic, antibacterial, and insecticidal activity.

Camellia shells can be used to prepare high-quality activated carbon and electrodes. The shell activated carbon is a porous carbon material generated through the carbonization and activation process that is widely used in decolourization and water purification. The shell is a residue produced during the production process and is not effectively utilized; making full use of this waste to prepare high value-added products should thus be a major focus of future research.
